# Intraoperative Assessment of Nerve Traction Injury in Obstetric Brachial Plexus Palsy

**DOI:** 10.1055/a-2572-2601

**Published:** 2025-05-02

**Authors:** J. Bahm, B. Schäfer, K. Nolte, J. Weis, J.P. Beier

**Affiliations:** 1 Division of Plexus Surgery, Department for Plastic Surgery, Hand Surgery - Burn Center, University Hospital RWTH, Aachen, NRW, Germany; 2 Institute of Neuropathology, University Hospital RWTH, Aachen, Germany

**Keywords:** obstetric palsy, brachial plexus, nerve traction, fibrosis, perineurium, endoneurium, minifascicles, nerve regeneration

## Abstract

We present an easy classification for nerve lesions observed in reconstructive surgery for obstetric brachial plexus palsy, performed through a supraclavicular approach and systematic exposure of nerve roots and trunks. A description of signs related to nerve traction injury (scarring, fascicular rupture, and dislocated ganglions) is combined with a grading system of microscopic tissue changes occurring in slices from traumatized nerve endings (fascicular structure, changes in perineurium and endoneurium). Both tools are proposed for any surgical brachial plexus exploration and later interaction with other professionals (pediatricians, physiotherapists, or obstetricians).

## Introduction

Obstetric brachial plexus palsy (obpp) is a rare complication of difficult delivery. 1 2 There is agreement among brachial plexus surgeons that traction on nerve structures determines the extent and severity of the lesion, and that cases with insufficient functional recovery should undergo reconstructive surgery.

Brachial plexus exploration is routinely conducted using a supraclavicular approach, allowing extensive intraoperative diagnosis regarding soft tissue damage, scarring (fibrosis), and damage to the nerve roots and trunks of the brachial plexus.

Although a useful clinical (and preoperative) classification into four groups exists, 3 amended by a subdivision of group 2 lesions regarding early recovery of wrist extension, 4 and allowing a gross prognostic estimation, so far no systematic approach to the intraoperative description of the traumatized brachial plexus has been implemented.

Regarding any clinical assessment, the Narakas classification ( Table 1 ) 3 5 assigns patients to four groups, according to the involvement of spinal nerve roots in the visible palsy pattern.

**Table 1:** Narakas classification

Group	Description
1	C5C6 (Erb)
2	C5C6C7 (extended Erb)
3	Total without Horner
4	Total with root avulsions

Group 1: Erb's palsy, includes injury to the C5 and C6 nerve roots and weakness of shoulder abduction, external rotation, and elbow flexion.
Group 2: the extended Erb's palsy, involves injury to the C5, C6, and C7 nerve roots, resulting in weakness of wrist and elbow extension in addition to the weakness experienced by patients in Group 1.
Group 3: a total obpp without Horner's syndrome, involves injury to the C5 through T1 nerve roots and total paralysis of the affected arm.
Group 4: a total obpp combined with Horner's syndrome, usually involves nerve root avulsion(s).

The Narakas classification does not include Klumpke's palsy, a syndrome of isolated hand weakness caused by injury to the lower nerve roots. Klumpke's palsy is very rare, only occurring in approximately 0.6% of patients with obpp. 6

### Our Macroscopic and Microscopic Intraoperative Assessment

In addition to this clinical and preoperative classification, we propose a simple, straightforward intraoperative classification of the surgically exposed nerve lesion ( Table 2 ). The idea is to classify intraoperatively observed lesions according to the severity of nerve damage and topography. On the one hand, this should provide the basis for an even clearer presentation and communication of intraoperative findings. This facilitates the possibility of discussion and better follow-up of patients, which can finally be helpful in a more precise assessment and communication of a prognosis. In addition to communication with the patient, this is also helpful in exchanges with other disciplines such as pediatricians or therapists to avoid unrealistic expectations regarding the reconstruction.

**Table 2:** Extent and severity of nerve lesions visible at supraclavicular exposure

Grade	Description
1	Minor interscalenic scarring, mostly affecting C5 and C6 roots and the emerging suprascapular nerve, without neuroma formation
2	Classical C5 C6 upper obpp with neuroma in continuity (Erb)
3	Extended upper obpp, including also C7, usually with conglomerate neuroma of the upper and middle trunk
4	Global lesions including the lower trunk
5	No supraclavicular scarring or signs of nerve injury, but electrophysiological signs of (partial) avulsion of upper roots (C5–C7), frequent in breech presentation
(Dis)	Dislocation of nerve structures, like the extraforaminal presence of spinal ganglia, distalisation of the suprascapular nerve, retro- or infraclavicular displacement of major nerve trunks

**Fig. 1:**
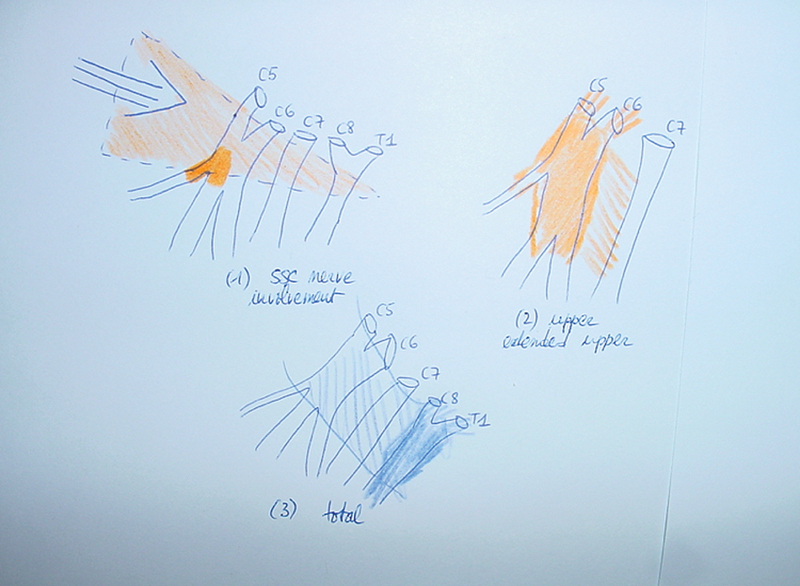
Translation of external lateral traction forces between head and shoulder onto the nerve structures of the brachial plexus: ( **1** ) The most lateral structures are first involved, giving rise to superficial scarring and involvement of the upper trunk and especially the origin of the suprascapular nerve. ( **2** ) A more severe force affects the upper and eventually middle trunk, responsible for Erb́s or extended Erb́s palsy. ( **3** ) Major traumatization spreads to the whole brachial plexus, with the most severe damage (including root avulsions) of the lower trunk (in deep blue, bold).

Bearing in mind that a nerve stretch appears when the head–neck area and the shoulder region are bent in opposite directions, the disruptive force is working first on outer (lateral) structures like the suprascapular (SSC) nerve and upper trunk, before gaining more inner (medial) structures like the middle and lower trunk ( Fig. 1 ).

In addition to this topographic classification ranging from 1 to 5, the appendix “dis” (displacement) is noted if one clearly observes a dislocation of anatomical landmarks. Examples are the extraforaminal presence of spinal ganglia, either isolated or within a conglomerate neuroma; the distalisation of the SSC nerve or of main nerve trunks which may be found behind or distal to the clavicle (in the retro- or infraclavicular space), requiring in rare situations a separate, deltopectoral approach.

In our institution, this descriptive classification is regularly complemented by a microscopic grading system applied to all nerve segments either proximal or distal to the neuroma, resulting from intraoperative (frozen sections) and postoperative (paraffin and resin section histology) neuropathological examination ( Table 3 ).

**Table 3:** Neuropathologic grading of traumatic nerve samples

Grade	Description
A	Preservation of fascicular nerve structure
	A1: normal, A2: minor disruption, A3: moderate disruption, A4: complete disruption
B	Endoneural fibrosis/inflammation
	B1: none, B2: minor, B3: moderate, B4: prominent
C	Perineurium
	C1: normal, C2: slightly thickened, C3: moderately thickened, C4: prominently thickened
D	Axonal loss
	D1: none, D2: minor, D3: moderate, D4: prominent
E	Neuroma formation
	E1: none, E2: minor, E3: moderate, E4: prominent
F	Presence of ganglion cells
	+ Indicative of root avulsion and − probably no avulsion

We thereby analyze first the macroscopic preservation of the fascicular structure in such big (root and trunk) nerves, the amount of connective tissue/collagen (signing local fibrosis) and inflammation within the endoneurium, the perineurial shape, axonal loss and neuroma formation (presence of minifascicles). The presence of ganglion cells within the examined slice signs a dislocated spinal ganglion, out of the foramen, which means that the connection to the myelon is fragilized and thus the observed root area is no longer in a safe connection to the myelon, thus excluding any grafting from this root.

**Fig. 2:**
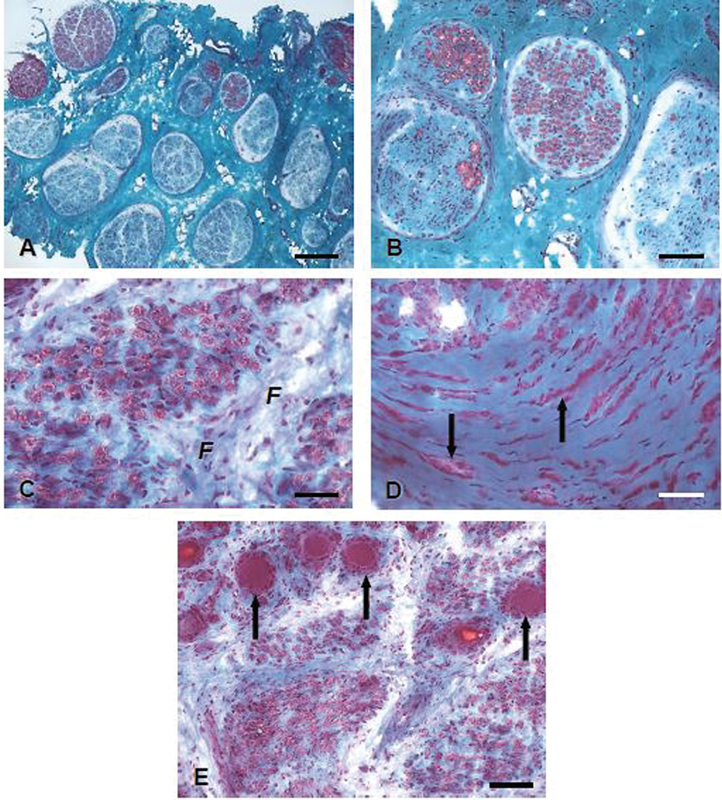
Neuropathological tissue changes affecting fascicular structure, endo-, and perineurium.

An example is provided in the illustrations collected in Fig. 2 .

This additional neuropathological information has been collected in our institution since 2000 and depends on the availability of specialized, goal-trained pathologists with expertise in traumatic neuropathology.

We are aware that this expertise is actually not available in many brachial plexus programs all over the world and that the described pathological patterns may not be so far clearly correlated to the quality of functional recovery. Never theless, we see the classification presented here as an opportunity to correlate histopathological results and clinical findings and to work out possible correlations. This classification, which we have not yet used systematically and which is based on clinical experience, offers a good opportunity to do this.

### Application

The aim of both classifications is to achieve an easily applicable, yet comprehensive description of the macro- and microscopic aspects of nerve traction injury and to provide a means of promoting discussion of the physical reality of mechanical distension injury with obstetricians and other health care providers like pediatricians and physiotherapists, who are the primarily involved caregivers in newborns affected by obpp.

It should be mentioned that grade 1 in our clinical routine with the expected picture of a lack of external rotation of the shoulder with otherwise good recovery of the arm function would not lead to an exploration of the brachial plexus. In these cases, we would normally prefer a selective nerve transfer of the partial spinal accessory nerve to the suprascapular nerve via a dorsal approach. Intraoperative assessment of the plexus is therefore not possible and not necessary. A grade 1 classification should therefore be assigned to these cases.

Furthermore, as both classifications are simple and comprehensive, their implementation may be progressively foreseen in other centers, first by the brachial plexus surgeon who classifies his intraoperative observation, and next by his colleague specialized in neuropathology who will look at the microscopic tissue changes. Thereby, both tools will enhance knowledge and exchange of information among dedicated brachial plexus surgeons.

Moreover, we strongly believe that grafting target nerves onto rather healthy areas of donor roots should provide better functional reinnervation.

Finally, their clinical usefulness will depend on the large spread of this information and a permanent application of the described criteria, altogether with a continuous reappraisal of the described neuropathological signs and further study of their prognostic correlation to clinical outcome.

The aim of both classifications, especially the histologic classification, cannot and must not be that each of the treating physicians involved knows the complete details. Rather, the aim should be that, starting from the surgeon, who can determine the intraoperative classification and who can thus supplement the histopathologic classification, realistic prognosis estimates can be given to all co-treating disciplines. This can only succeed if, based on standardized classifications, further studies are performed looking at the clinical outcome and possible correlations.
